# Structure Development of the Interphase between Drying Cellulose Materials Revealed by In Situ Grazing-Incidence Small-Angle X-ray Scattering

**DOI:** 10.1021/acs.biomac.1c00845

**Published:** 2021-09-20

**Authors:** Hailong Li, Stephan V. Roth, Guillaume Freychet, Mikhail Zhernenkov, Nadia Asta, Lars Wågberg, Torbjörn Pettersson

**Affiliations:** † Department of Fibre and Polymer Technology, KTH Royal Institute of Technology, Teknikringen 58, SE-100 44 Stockholm, Sweden; ‡ Department of Physics, AlbaNova University Center, Stockholm University, Stockholm 10691, Sweden; § Deutsches Elektronen-Synchrotron (DESY), Notkestr. 85, Hamburg 22607, Germany; ∥ National Synchrotron Light Source II, Brookhaven National Laboratory, Upton, New York 11973, United States; ⊥ Wallenberg Wood Science Centre, Department of Fibre and Polymer Technology, KTH Royal Institute of Technology, Teknikringen 56, Stockholm 10044, Sweden

## Abstract

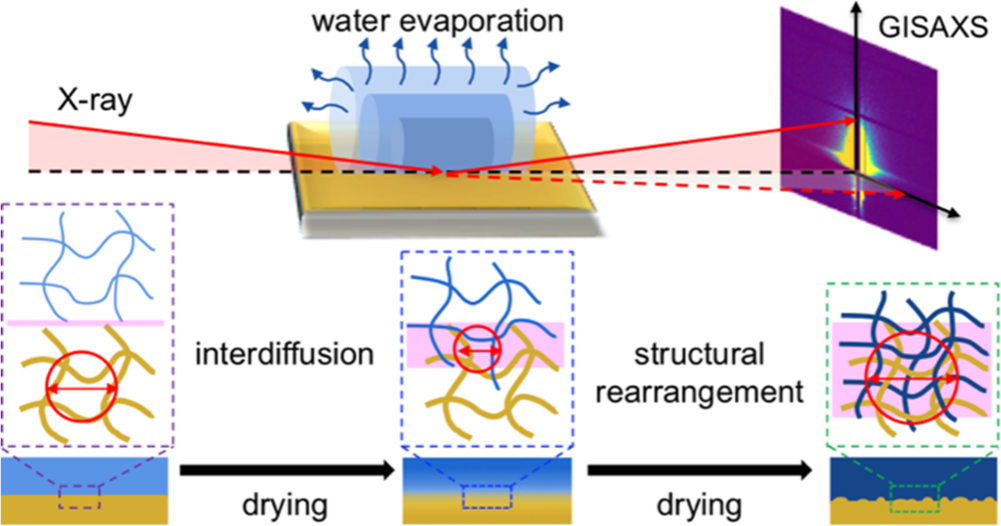

The nano- to microscale structures at the interface between materials can define the macroscopic material properties. These structures are extremely difficult to investigate for complex material systems, such as cellulose-rich materials. The development of new model cellulose materials and measuring techniques has opened new possibilities to resolve this problem. We present a straightforward approach combining micro-focusing grazing-incidence small-angle X-ray scattering and atomic force microscopy (AFM) to investigate the structural rearrangements of cellulose/cellulose interfaces in situ during drying. Based on the results, we propose that molecular interdiffusion and structural rearrangement play a major role in the development of the properties of the cellulose/cellulose interphase; this model is representative of the development of the properties of joint/contact points between macroscopic cellulose fibers.

## Introduction

Natural raw materials such as protein, silk, or wood have attracted increasing attention in the development of environmentally friendly products.^1−5^ Cellulose, mainly extracted from plants, has gained particular attention owing to its natural abundance, renewability, low cost, biodegradability, and excellent mechanical properties.^6−9^ On an industrial scale, it has been converted into paper, packaging materials, filaments, and textiles; it is also being used as a carrier in chromatography, separation technology, and life science applications.^10−17^ The mechanical properties of these materials are strongly determined both by the supramolecular structure of cellulose and the molecular interaction between the cellulose surfaces and other materials, especially in the wet state where the joints are developed and then consolidated during drying.^4,5^ Despite extensive research in this field, structural information of the molecular processes controlling these interactions is very limited.^18,19^ This is partially due to the lack of well-characterized cellulose model surfaces and partially due to the lack of high-resolution measurement techniques capable of characterizing the structure of the interfaces and interphase throughout the joining process. Before the joining process, an interface exists between two distinct phases, while an interphase, which is the intermediate phase between two phases,^20^ will form during the joining process in the composite.

Recently, we have fabricated millimeter-sized cellulose gel beads as model surfaces by precipitating cellulose/lithium chloride (LiCl)/*N*,*N*-dimethylacetamide (DMAc) solution into a nonsolvent (ethanol or water).^21−25^ These gel beads have been used as a model system for investigating the swelling behavior of the wet, delignified wood cellulosic fiber wall.^24^ Nuclear magnetic resonance and small-angle X-ray scattering investigations of the gel beads indicated that their internal structure can be considered as a homogeneous, noncrystalline, and molecularly dispersed polymer network.^23,24^ However, to our knowledge, there is no study available that characterizes the microstructural changes at the interface of two cellulose surfaces when drying in contact with one another. This is an important fundamental process to understand as it defines the physical properties of the majority of the products made from cellulose. Since grazing-incidence small-angle X-ray scattering (GISAXS) was first used to study thin film growth by Levine et al. in 1989,^26^ it has been successfully utilized to probe the structures of thin films formed at liquid^27,28^ or solid surfaces.^29−34^ The structures of different cellulose thin films and the water-induced structural rearrangements of these cellulose films^35−37^ have also been resolved by GISAXS measurements. Although GISAXS has been successfully applied to these different research areas, its application to study liquid/solid and liquid/liquid interfaces is quite rare.

**Figure 1:**
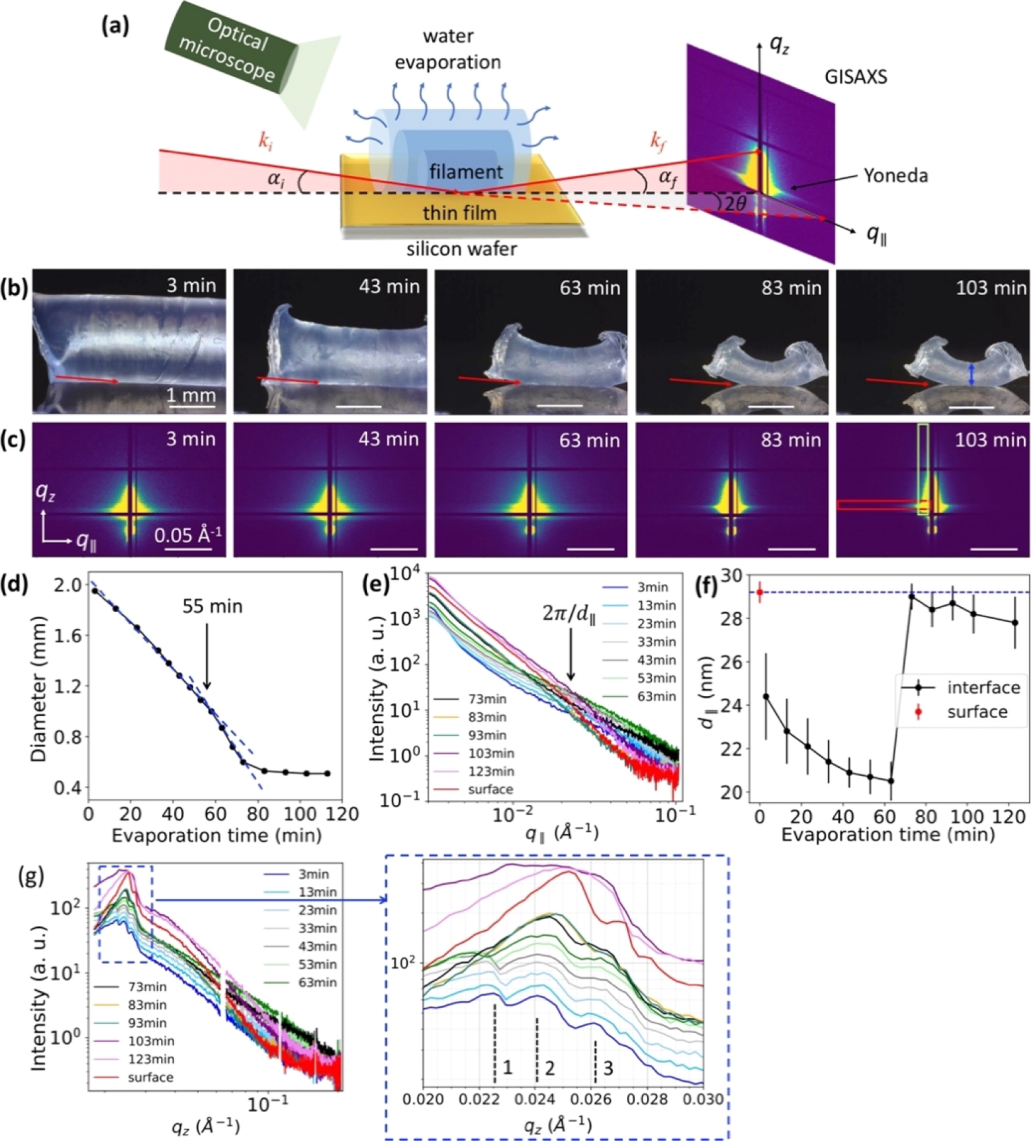
(a) Schematic illustration of the in situ μGISAXS measurements during drying of the water-swollen filament on a cellulose surface. *q*_∥_ and *q*_*z*_ are the components of the wavevector transfer parallel and vertical to the sample surface, respectively (details can be found in the Methods section). An optical microscope was also used to monitor the size of the filament during drying. (b) Representative microscope images of the cellulose filament were collected after different drying times. The red arrows indicate the incident X-ray direction. The scale bar corresponds to 1.0 mm. (c) 2D μGISAXS patterns of the interface between the cellulose filament and cellulose thin film at the corresponding evaporation time shown in (b). The red and green boxes represent the areas that were integrated for extracting the horizontal and vertical cuts, respectively. The scale bar corresponds to 0.05 Å^–1^. (d) Diameter vs evaporation time curve of the filaments as measured from microscopy images. The diameter was measured at the same position on each filament as indicated by the blue double-headed vertical arrow in the far-right image in (b). (e) Horizontal cuts of the 2D μGISAXS patterns obtained at different drying times. (f) Average distance *d*_∥_ vs evaporation time curve calculated from curves in (e). (g) Vertical cuts of the 2D μGISAXS patterns obtained at different drying times. The red curves in (e,g) and the red points in (f) are controls from measurements of the dry thin film surface without the filament.

In the present study, designed to resolve the microstructural change of a cellulose/cellulose interphase during drying, micro-focusing GISAXS (μGISAXS) experiments were performed using cellulose model surfaces. To achieve a large interfacial area enabling μGISAXS measurements, millimeter-sized water-swollen cellulose gel filaments were fabricated (Figure S1 in Supporting Information). Cellulose thin films were spin-coated onto silicon wafers according to our previous protocol (Figure S2) and were used as contacting surfaces for the filaments.^38−40^ The in situ μGISAXS measurements were performed to analyze the interphase between the cellulose filaments and the cellulose thin film (Figures 1a and S3).

## Experimental Section

### Materials

Domsjö dissolving pulp (Domsjö Fabriker AB, Sweden) was used as the raw material to prepare the cellulose/DMAc/LiCl solution, gel beads, and gel filaments. The fibers from this dissolving pulp contain 96% glucose,^23−25,41^ and their charge density is reported as 29 μeq/g.^42^ LiCl (puriss p.a., anhydrous ≥99%), DMAc (puriss p.a., ≥99.5%), *N*-methylmorpholine-*N*-oxide (NMMO, 50 wt % solution in water), DMSO (99%), sodium hydroxide (NaOH, puriss p.a. ACS reagent ≥98%), and ethanol (96 vol %) were purchased from Sigma-Aldrich and used as received. Polyvinyl amine (PVAm) was sourced from the commercial product Lupamin 9095, provided by BASF in a water solution with a reported total solid concentration of 20–22 wt % and pH = 7–9.

### Preparation of Cellulose/DMAc/LiCl Solution

In order to make water-swollen cellulose filaments and cellulose beads, cellulose/DMAc/LiCl solution was first prepared according to the previously published protocol.^21,23−25,43^ The dissolving pulp was prewashed with deionized water to remove metal ions and dissolved colloidal substances (carbohydrates, lignin, and extractives). The water-saturated dissolving pulp containing 1.5 g of dry mass was subjected to solvent exchange first to ethanol and subsequently to DMAc through multiple washing/filtration steps. The solvent exchange was performed over 2 days for each solvent, the solvent being changed at least twice per day using 150 mL each time. After the solvent exchange, 100 mL of DMAc was heated to 105 °C for 20 min in an oil bath, and 7 g of LiCl was heated in an oven at 105 °C for 30 min to dry. The dehydrated LiCl was added to the heated DMAc and then allowed to cool. When the temperature reached 65 °C in the DMAc/LiCl mixture, the DMAc-saturated pulp was added to the dehydrated DMAc/LiCl solution. After one night of stirring, transparent 1.5 wt % cellulose solution was obtained.

### Preparation of Cellulose Gel Filaments and Beads

The cellulose/DMAc/LiCl solution was injected into an antisolvent (ethanol) bath, where the cellulose solution solidified as a cylindrical filament, as shown in Figure S1. The prepared cylindrical filament was left to equilibrate for 24 h in the ethanol bath. Subsequently, it was continuously washed with fresh Milli-Q water for at least 7 days to ensure the proper removal of the cellulose solvent to leave a Milli-Q water-swollen cellulose filament.

A nearly identical precipitation protocol was used to prepare cellulose gel beads. The only difference was that the cellulose/LiCl/DMAc solution was precipitated dropwise into the ethanol bath to form beads which were left to equilibrate for 1 day. The beads were then washed with fresh Milli-Q water or ethanol for more than 7 days to obtain water-swollen or ethanol-swollen beads.

### Preparation of Cellulose Thin Films

Cellulose thin films were spin-coated on silicon wafers by following the protocol published by Gunnars et al.,^38^ Eriksson et al.,^39^ and Benselfelt et al.^40^ A cellulose/NMMO/DMSO solution was first prepared by dissolving 0.25 g of dry pulp in 12.5 mL of NMMO (50 wt % in water) at 125 °C under magnetic stirring for approximately 60 min (see Figure S2). At which point a clear solution was obtained, 37.5 mL of DMSO was added dropwise to dilute the solution for the spin-coating process.

The silicon wafer was first washed with a sequence of water, ethanol, and water, dried with N_2_ gas, and oxidized at 1000 °C for 1 h, during which a less than 100 nm thick SiO_2_ layer was formed. The oxidized wafers were hydrophilized by submersing in 10 wt % NaOH solution for 60 s before rinsing and drying with N_2_ gas and placed under plasma (PCD 002, Harrick Scientific Corp., Ossining, NY, US) for 3 min. After that, the wafer was dipped in PVAm solution (0.1 g/L, pH 7.5) for 15 min to adsorb a PVAm layer onto which the cellulose film could later be anchored; it was then rinsed with Milli-Q water and dried with N_2_ gas.

The aforementioned cellulose/NMMO/DMSO solution (at 125 °C) was spin-coated on the PVAm-treated oxidized silicon wafer at 1500 rpm for 15 s, followed by 3500 rpm for 30 s using a spin coater (KW-4A-2, Chemat Technology, Northridge, CA, USA). Then, the spin-coated thin films were subjected to solvent exchange in Milli-Q water for 12 h, after which the water was exchanged three times every 1 h and dried with N_2_ gas. Finally, the cellulose thin films were cured at 105 °C overnight to improve the wet stability.

### In Situ μGISAXS Measurements

The in situ μGISAXS measurements were carried out for the cellulose filament/thin film interface at the soft matter interfaces (SMI, 12-ID) beamline at the National Synchrotron Light Source II (NSLS-II) in Brookhaven National Laboratory.^44^ The experiments were conducted using an incident photon energy of 16.1 keV (wavelength λ = 0.77 Å) with a beam size of 2.3 × 25 μm (vertical × horizontal, see Figure S3a,b). The 2D μGISAXS patterns were recorded using a Pilatus 1 M detector (981 × 1043 pixels, pixel size: 172 × 172 μm). The sample-to-detector distance was 6.2 m. The X-ray incident angle (α_i_) was set to α_i_ = 0.1°. The length of the incident beam footprint was calculated to be 2.3 μm/sin (0.1°) = 1.3 mm. Under these conditions, the scattering intensity from a surface area of 1.3 mm × 25 μm (length × width, see Figure S3b) could be collected.

1D intensity profiles were extracted from 2D μGISAXS patterns as a function of the scattering vector *q*, which is composed of its single components *q*_*x*_, *q*_*y*_, and *q*_*z*_. They can be written as^45^
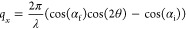
1
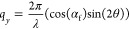
2
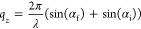
3where λ is the wavelength, α_i_ and α_f_ are the incidence and exit angle, respectively, and 2θ is the out-of-plane angle with respect to the scattering plane spanned by *k*_i_ and *k*_f_. Throughout the rest of this work, we use  and *q*_*z*_ reciprocal planes to describe scattering, reflection, and refraction parallel and perpendicular to the sample surface.

For the μGISAXS measurements, a wet cellulose gel filament with roughly 2.0 mm diameter was placed on the silicon wafer, which was spin-coated with a cellulose thin film. The excess water from the wet cellulose gel filament allows the thin film to be wetted and later re-dried together with the filament. To track the position of the filament/thin film interface, optical microscopes were used to quickly locate the center of the interface. The accurate *X* and *Z* positions of the interface were obtained through a *Z*-scanning method, as shown in Figure S3a,b. The motorized stage, on which the sample was placed, moved relatively from −20 to +20 μm with 41 steps in the *Z* direction (Figure S3a) to make sure the X-ray footprint (length × width: 1.3 mm × 25 μm) can scan over the whole thin film surface in the *X* direction (red rectangular area shown in Figure S3b). At each moving step, the sample was exposed to an X-ray beam for 0.5 s, and one 2D μGISAXS pattern was obtained. Thus, 41 2D μGISAXS patterns were obtained for one *Z*-scanning. The same *Z*-scanning was performed on the thin film surface as indicated in the light green rectangular area in Figure S3b.

Two of the 2D μGISAXS patterns during the *Z*-scanning are shown in Figure S3c,d for the filament/thin film interface and thin film surface, respectively. Integrating the 2D q range, as indicated by the red rectangular area shown in Figure S3c, for all 41 2D μGISAXS patterns gives the intensity profile during *Z*-scanning for the interface or surface, which were plotted, as shown in Figure S3e. It was observed that the X-ray beam shot the center of the filament/thin film interface when the scan step was “19” throughout the entire drying process. The 2D μGISAXS patterns of the filament/thin film interface and thin-film surface at scan step 19 (Figure S3c,d) exhibit clear differences. This will be further discussed below. All the 2D μGISAXS patterns and their corresponding 1D intensity profiles used in the main text were obtained at scan step 19.

During the drying process, the *Z*-scanning was repeated at the filament/surface interface every 10 min. After each *Z*-scanning, the sample was shifted 10 μm in the *Y* direction to avoid overshooting the sample, which can cause radiation damage.

### Pulling off Measurements

The mechanical test was conducted by a micro-adhesion measurement apparatus, also known as a contact adhesion tester, which has been used to detect adhesion forces in previous studies.^25,39,46,47^ To simplify the mechanical pulling off experiments, cellulose gel beads were used instead of cellulose filaments. The cellulose gel beads were dried on a cellulose thin film (which was itself dry before having gel beads placed on it). A top plate, coated with thin epoxy glue, was lowered onto the surface of the dried beads. After curing the epoxy glue for 1 h, unloading started at a rate of 10 μm/min until the beads were pulled off the thin film surface. For comparison, the same measurements were performed for beads that were first dried on Teflon and then placed on the cellulose thin film.

### Macro- and Micromorphology Characterization

The diameter changes of the cellulose filament, as shown in Figure 1b, were monitored during the entire drying process using an optical microscope (AM7013MZT, Dino-Lite Premier Digital Microscope). Other optical microscope images were acquired for the contact area of cellulose beads and thin films after separation using an optical reflection microscope (Olympus U-TVO.5X; Olympus Optical Co., Tokyo, Japan). SEM images were taken by using a high vacuum S-4800 field emission scanning electron microscope (Hitachi, Tokyo, Japan). The surface roughness and morphologies of the prepared cellulose thin films, dried beads, and the corresponding edges of the interface after separating the beads from thin films were measured by an atomic force microscope MultiMode 8 (Bruker, Santa Barbara, CA, USA) setup using the SCANASYST mode with a SCANASYST-AIR cantilever. They were measured in the dry state in the air under ambient conditions.

## Results and Discussion

### Microstructures of the Cellulose Interphase during Drying

Figure 1b shows that the filament shrinks continuously with increasing drying time, while the cylindrical cross section is retained throughout the evaporation process. However, after roughly 55 min of drying, the filament starts to bend up. The diameter of the cellulose filament during the drying process was plotted, as shown in Figure 1d. The diameter decreases from 1.95 to 1.05 mm during the first 55 min and then decreases slightly faster to 0.52 mm over the next 25 min; following this, the diameter remains constant. A similar drying behavior was previously observed for drying water-swollen cellulose gel beads,^21,22^ suggesting that the drying kinetics of the filament is the same as that of the bead. Note that the cellulose thin film remains flat during drying, and the bottom filament seen in the microscopic images is the reflection of the filament.

Figure 1c shows the corresponding 2D μGISAXS patterns of the filament/thin film interface. The μGISAXS pattern changes drastically between 63 and 83 min, both in the *q*_*z*_ and *q*_∥_ direction, which indicates sharp structural changes in the interphase during this drying period. After that, the μGISAXS pattern stabilizes and does not change anymore. According to our previous in situ wide-angle X-ray scattering investigation of the cellulose gel bead (prepared from the same cellulose solution as in this work), barely any crystallization was observed during the drying process.^21^ This excludes the influence of crystallization on the μGISAXS experiments. To further analyze the correlation between the macroscopic diameter change and the microstructural change, we performed two distinct integrations to all the 2D μGISAXS patterns, a horizontal integration (red rectangle: 0.003 Å^–1^ ≤ *q*_∥_ ≤ 0.11 Å^–1^ and 0.023 Å^–1^ ≤ *q*_*z*_ ≤ 0.028 Å^–1^) and a vertical integration (green rectangle: 0.017 Å^–1^ ≤ *q*_*z*_ ≤ 0.21 Å^–1^ and 0.005 Å^–1^ ≤ *q*_∥_ ≤ 0.018 Å^–1^). The intensity versus *q*_∥_ and *q*_*z*_ curves are presented in Figure 1e,g, respectively. The evolution of the scattering profiles versus the evaporation time for both graphs indicates a structural change during drying.

The occurrence of a side peak in the *q*_∥_ plots shown in Figure 1e, indicated by the arrow, allows for the determination of a characteristic in-plane length scale via *d*_∥_ = 2π/*q*_∥_,^29,48^ where *d*_∥_ is attributed to the average distance between cellulose aggregate structures. A plot of *d*_∥_ versus evaporation time is shown in Figure 1f. The data show that *d*_∥_ changes from 29.2 ± 0.5 to 24.4 ± 2.0 nm after placing the water-swollen cellulose gel filament onto the cellulose thin film. The reduction of *d*_∥_ is probably caused by capillary forces and the interaction between the wet gel filament and the dry thin film, and alternatively, it may be caused by the diffusion of water or still mobile cellulose molecular chains from the outermost layer of the gel filament into the thin film. During the first 60 min of evaporation, *d*_∥_ steadily decreases to 20.5 ± 0.9 nm, after which a sharp increase of *d*_∥_ to 29 ± 0.6 nm was observed. The sharp increase of *d*_∥_ is linked to the structural change of cellulose which was observed in our previous SAXS study.^21^ In the late drying phase, a slight decrease of *d*_∥_ with evaporation time was detected. We note that the steep increase occurs at approximately the same time as the macroscopic diameter levels off.

The vertical cuts presented in Figure 1g show correlations orthogonal to the surface of thin film, which includes the strongest scattering, the so-called “Yoneda peak”, and resonant diffuse scattering.^30,31,49−51^ A zoom-in image of the region around the Yoneda peaks is presented to the right of Figure 1g. Before placing the filament on the dried cellulose thin film, the Yoneda peak of the dried cellulose thin film was observed at *q*_*z*_ = 0.0253 Å^–1^ (Peak 2, red curve) (details in Supporting Information). The oscillations at higher *q*_*z*_s, for example, Peak 3, are attributed to the resonant diffuse scattering peak due to thin film interference, which indicates that a very smooth cellulose thin film was prepared. After placing the water-swollen filament onto the thin film, it is observed that the *q*_*z*_ profile in the Yoneda region changes significantly. This is mainly due to the fact that the gel filament becomes the top subphase (Figures S4 and S5). The newly appeared peak at *q*_*z*_ = 0.0225 Å^–1^ (Peak 1) can be attributed to the presence of a new layer at the surface of the cellulose thin film, which we refer to as the interphase layer (Figure S4c). It most probably arises from the diffusion of the most mobile cellulose molecular chains from the outermost layer of the gel filament into the thin film. This is consistent with the sharp decrease of *d*_∥_ after the same drying time, as shown in Figure 1f. Between 3 and 63 min of drying, all three peaks start to broaden and shift to the right. This indicates that the roughness of the interphase layer is continuously increasing due to the continuous diffusion of cellulose molecular chains during this interval. Peaks 1 and 3 can no longer be resolved after drying for 73 min (black curve shown in Figure 1g), and Peak 2 shifts back to the position of the initial dry film. This is attributed to the swollen filament that starts to partially detach from the cellulose thin film (Figure 1b). Consequentially, the filament cannot be considered an effective subphase beyond this time point. In the later drying phase, from 103 min on, Peak 2 becomes broader and the intensity drop shifts to a higher *q*_*z*_ than the initial dry thin film surface. This indicates the presence of a denser and rougher layer at the interface after drying, hypothetically caused by the structural rearrangement of cellulose chains in the interphase layer. These results suggest that two processes take place on the molecular level during water evaporation: interdiffusion and a structural rearrangement of the cellulose molecular chains at the interface.

**Figure 2:**
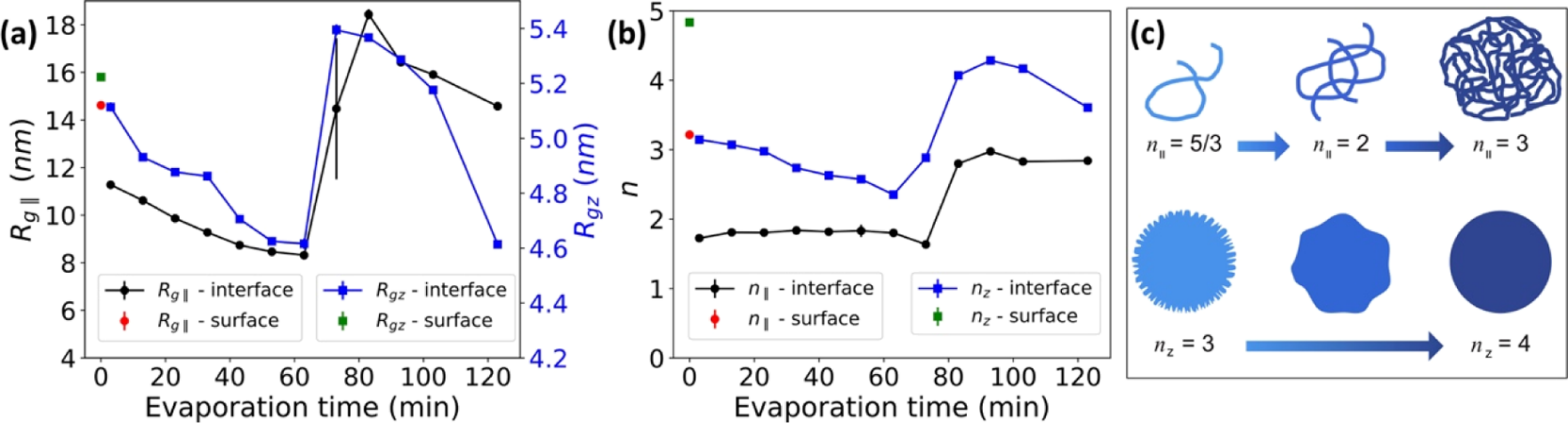
Time-dependent changes (blue and black curves) in the cellulose/cellulose interface caused by evaporation for (a) fitted length scale parameters (*R*_*g*∥_ and *R*_*gz*_) and (b) “dimensionality” parameters (*n*_∥_ and *n*_*z*_) of the horizontal and vertical cuts. The red and green symbols correspond to the dried cellulose thin film. (c) Specific value of parameter *n* and the corresponding shape of the object represented according to the literature.^52,53^*n*_∥_ = 5/3 and *n*_∥_ = 3 represent swollen and collapsed states of cellulose chains, respectively, while *n*_*z*_ = 3 and *n*_*z*_ = 4 indicate rough and smooth surfaces of objects, respectively.

In order to extract the parameters of the in-plane roughness (size and shape of the microstructures) along *q*_∥_ and *q*_*z*_, we used a Guinier-Porod model^52^ to fit the horizontal and vertical cuts, respectively. Although this is a simplified approach, it is reliable enough to indicate how the structure changes for this complex system. The fitting details are summarized in Supporting Information and Figure S6. The fitted characteristic length scale parameters *R*_*g*∥_ and *R*_*gz*_ and “dimensionality” parameters *n*_∥_ and *n*_*z*_ as a function of the evaporation time are plotted, as shown in Figure 2a,b, respectively. The size of the microstructures along *q*_∥_, which is determined from *R*_*g*∥_, decreases from 11.3 ± 0.2 to 8.3 ± 0.1 nm during the first 63 min of evaporation, and then, a sharp increase up to 18.4 ± 0.2 nm is observed over the next 30 min (from 60 to 90 min of drying). A similar larger increase of *R*_g_ was observed for the internal structure of the cellulose gel bead in the later drying stage due to the formation of cellulose aggregates.^21^ Finally, there is a decrease to 14.6 ± 0.1 nm after 123 min of drying. The size of the microstructures along *q*_*z*_ (*R*_*gz*_) follows a similar trend. *n*_∥_ and *n*_*z*_ exhibit a similar trend during drying, and the corresponding shapes are represented in Figure 2c. Thanks to the GISAXS technique, which gives nanoscale structure information at the interphase in both the vertical and horizontal directions, we interpret the results shown in Figure 2 as follows: (1) along *q*_∥_, the bulk structure of the interphase layer transfers from a swollen state (*n*_∥_ = 1.7) to a collapsed state (*n*_∥_ = 3.0) when the sharp increase *R*_*g*∥_ happens and (2) along *q*_*z*_, the objects (*R*_*gz*_ around 5 nm) change from spherical shapes with rough surfaces to spherical shapes with smooth surfaces due to the loss of water. However, on a microscopic scale, the roughness of the interphase still increases with continued drying, which is based on the results shown in Figure 1g.

### Adhesion Properties Between Cellulose/Cellulose Interface

**Figure 3:**
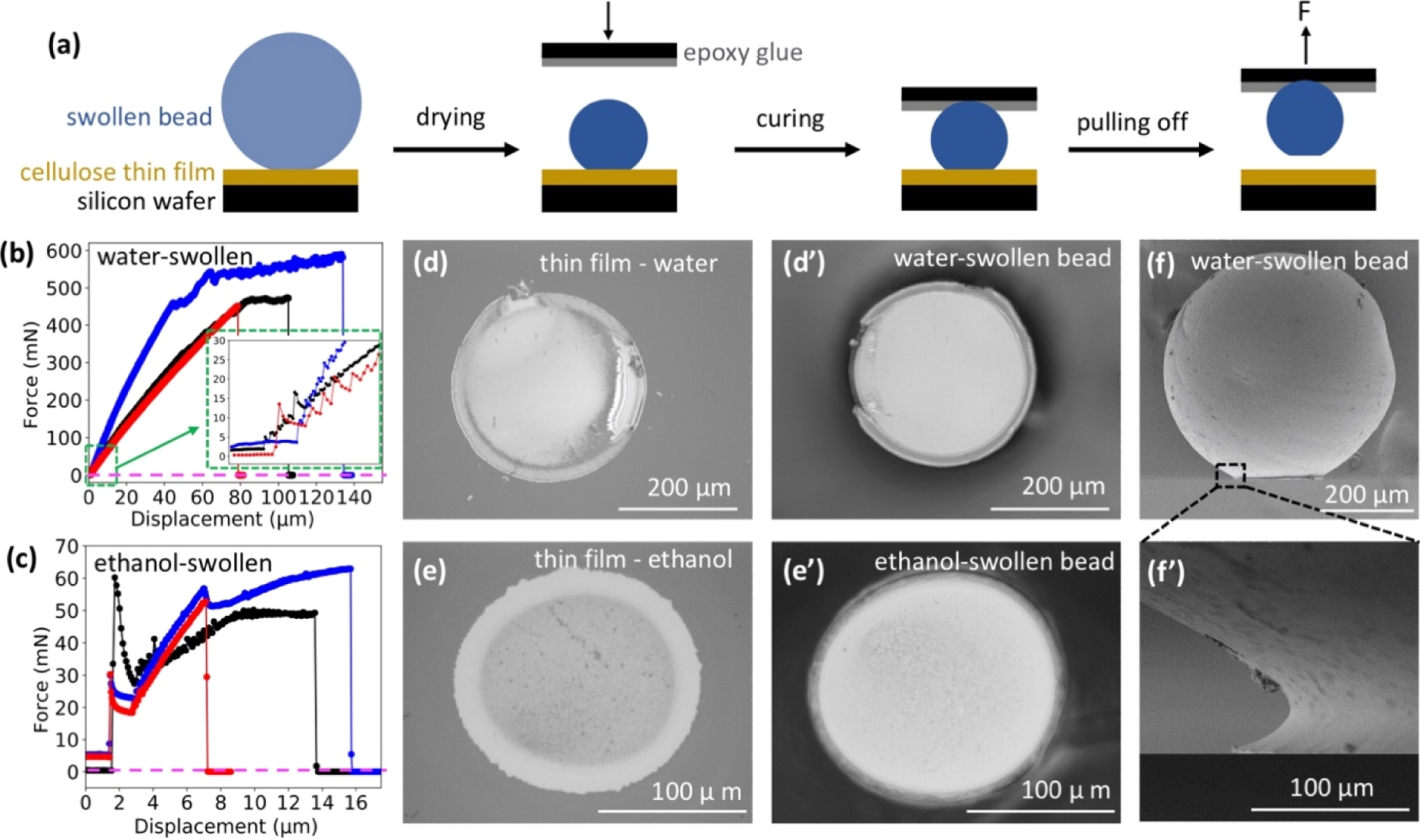
(a) Schematic illustration of the methodology behind the pulling off experiments. Three representative unload force vs displacement curves for pulling off the water-swollen (b) or ethanol-swollen (c) beads from the thin film after drying. The dashed lines (magenta) in (b,c) are controls obtained from pulling off beads that were first dried on Teflon and then placed on a cellulose thin film. Optical microscopy images of the thin film (d) and the bottom of the dried water-swollen bead (d′) after separation. Optical microscopy images of the thin film (e) and the bottom of the dried ethanol-swollen bead (e′) after separation. (f,f′) SEM images of dried water-swollen beads after 2 days of drying on the thin film.

To investigate the effect of the development of the molecular structure at the cellulose/cellulose interface on the macroscale adhesion between cellulose surfaces, mechanical testing was conducted by pulling off measurements of cellulose beads that had been allowed to dry on a cellulose thin film (Figure 3a). The average maximum separation force (Fs) was 500 mN for the water-swollen beads (Figure 3b); this is roughly 10 times larger than that for ethanol-swollen beads (55 mN on average, Figure 3c). Predried beads, which were first dried on Teflon and then placed on the thin cellulose film, were separated from the surface immediately when the pulling started, and no detectable force was needed to separate these beads from the surfaces (dashed lines shown in Figure 3b,c). This means that the adhesion of the beads to the cellulose thin film is dependent on processes that take place during the drying of the swollen beads. We postulate that this is due to the external molecular layers of the swollen cellulose beads integrating with the thin cellulose film during drying and thus forming the interphase layer that holds the beads and the thin film together after drying. This explanation is supported by the optical microscopy images of the contact zone of the thin films (Figure 3d,e) and the bottom of dried beads (Figure 3d′,e′) after separation from each other. When the water-swollen bead was pulled off, most of the contacted cellulose thin film came off of the silicon wafer with it (Figure 3d). Additionally, a concave shape was formed at the bottom of the water-swollen bead after drying, evidence of the merging of the two cellulose materials (Figure 3f,f′). In the same experiment conducted with ethanol-swollen beads, only a ring of the thin film was peeled off; naturally, a smaller separation force was recorded. By dividing the contact area by Fs, the pull-off stress (σ_s_) of the beads could be calculated; this assumes a complete molecular contact between the two surfaces. For the dried water-swollen beads, this was calculated to be 8.4 MPa, which is two times larger than that calculated for the dried ethanol-swollen beads (3.8 MPa). It is well known that water has higher interaction with cellulose compared to ethanol, and the attractive van der Waals interactions between cellulose and cellulose across a liquid medium are stronger in water compared to ethanol.^54^ Thus, the cellulose molecular chains on the outer surface layer of the water-swollen beads are much more mobile than those of the ethanol-swollen beads. This allows them to diffuse into and entangle more with cellulose in the thin film, forming a stronger cellulose interphase layer than the ethanol-swollen bead, which leads to high pull-off stress for dried water-swollen beads. This is consistent with the μGISAXS results shown in Figure 1g, which shows the development of a denser interphase layer for the water-swollen filament after drying. This interphase layer is sufficiently strong that it is able to survive the shrinking of the water-swollen beads during drying. Instead of detachment or breaking, a concave shape of the contact zone is observed (Figure 3f,f′).

### Morphologies of the Contact Area of Cellulose/Cellulose Interfaces

**Figure 4:**
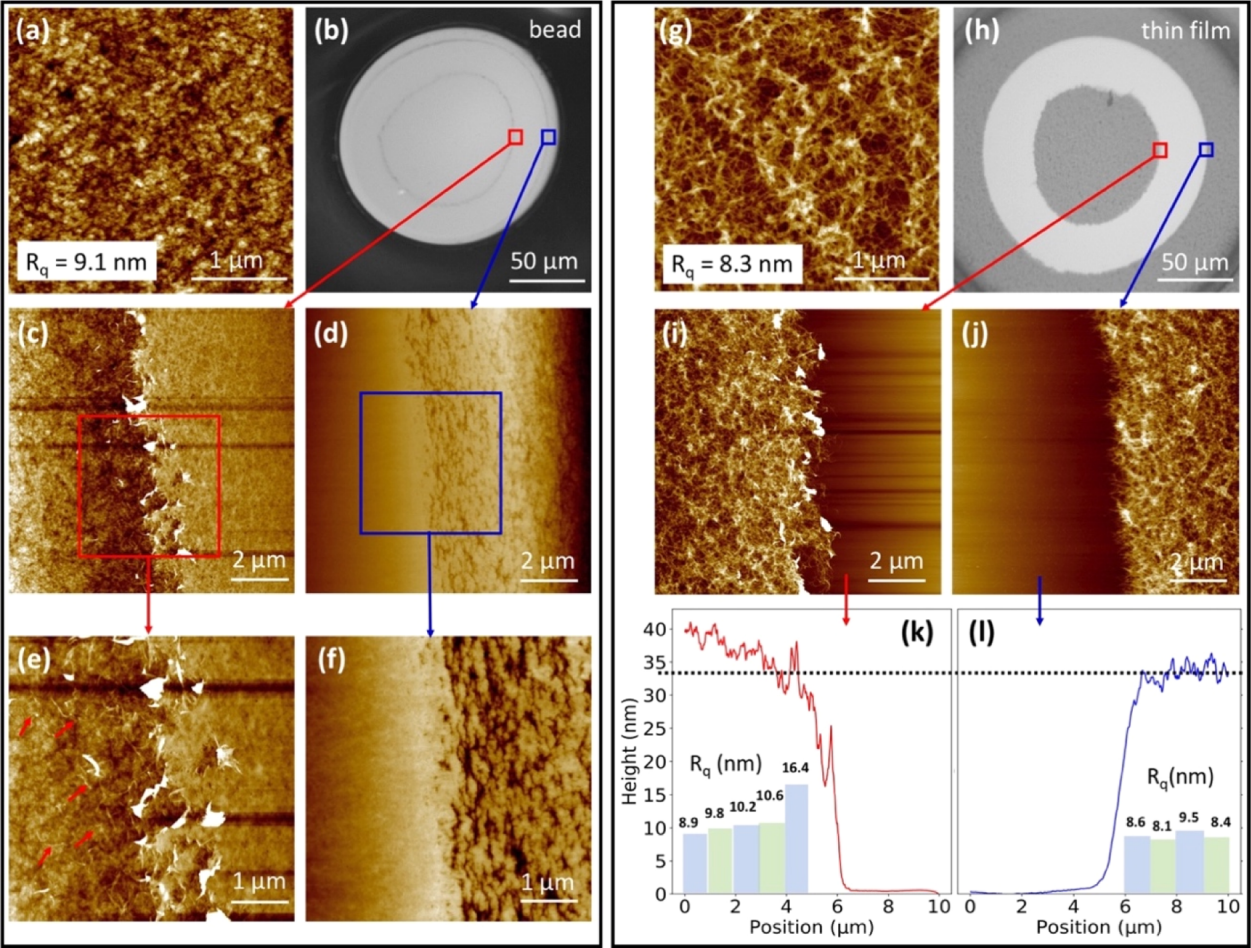
(a) AFM height image of the dried ethanol-swollen bead surface and its root mean square roughness (*R*_q_). (b) Microscopy image of the bottom of the cellulose bead after separation. AFM height images of the inner (c) and outer (d) edges of the attached ring from the cellulose film, and the corresponding high magnification AFM height images (e,f), respectively. The red arrows in (e) mark the cellulose fibrillar structures created when separating the surfaces from each other. (g) AFM height image of the dried cellulose thin film surface. (h) Microscopy image of the thin film at the contact area after separation from the dried ethanol-swollen bead. AFM height images of the inner (i) and outer (j) edges of the pulled off ring, and the corresponding average height curves (k,l), respectively. The dashed lines in (k,l) represent the thickness of the dried thin film. The charts and the corresponding indexes in (k,l) are the roughness *R*_q_ of the thin film calculated over every 1 × 10 μm^2^ (horizontal × vertical) region in (i,j).

Although the interphase layer formed with ethanol-swollen beads is much weaker, the reduced damage upon separation allows for the characterization of the morphologies of the contact area of both the thin film and the dried bead by AFM. Figure 4a–d,g–j shows that a ring of the cellulose thin film was peeled off the substrate and attached to the lower part of the dried bead. Similar results can be observed from the SEM images of a dried bead, as shown in Figure S7. A closer look at the inner edge of the ring in Figure 4e shows that the interphase layer consists of a homogenously distributed fibrillar structure from the thin film and the cellulose dried from the swollen bead. The observation of cellulose from the bead permeating the homogenously distributed fibrillar structure of the film is evidence of the diffusion of cellulose chains from the external part of the ethanol-swollen bead into the thin film (Figure 4g). Because the interphase layer formed with water-swollen beads is stronger than that formed by ethanol-swollen beads, it is reasonable to assume that more fibrillar structures should exist in the interphase layer formed by water-swollen beads. It was not possible to directly observe this due to the entire contact zone of the water-swollen beads peeling off the underlying silicon wafer. Interestingly, similar results have been detected for the adhesion between two polybutylmethacrylate surfaces,^55^ which strengthens the interpretation of the results in the present work. Furthermore, this theory is in keeping with the results of the μGISAXS experiments (Figure 1g), which indicate that a denser interphase is formed by the water-swollen beads upon drying. The height of the thin film adjacent to the final contact area after drying was consistently determined to be 33 nm, and its *R*_q_ was determined as 8.6 nm (Figure 4j). This is nearly identical to the *R*_q_ value of 8.3 nm measured on the thin film that had not been in contact with wet beads (Figure 4g). It can, therefore, be concluded that changes to the thin film are limited to those areas in direct contact with the swollen beads. Inside the contact area, the thin film increases in height to 40 nm (measured 5 μm inside the contact ring), and its *R*_q_ value decreases from 10.6 to 8.9 nm. A high roughness of 16.4 nm at the edge is caused by the fractured surface. Based on this data, we propose that cellulose chains from the swollen bead diffuse into and entangle with the 3D open network structure of the thin film. When pulling off the dried bead, the interphase layer is stretched and the roughness of the thin film inside the contact area increases (Figures 4k and S7d).

### Mechanistic Insights into the Adhesion of Cellulose/Cellulose Interfaces

**Figure 5:**
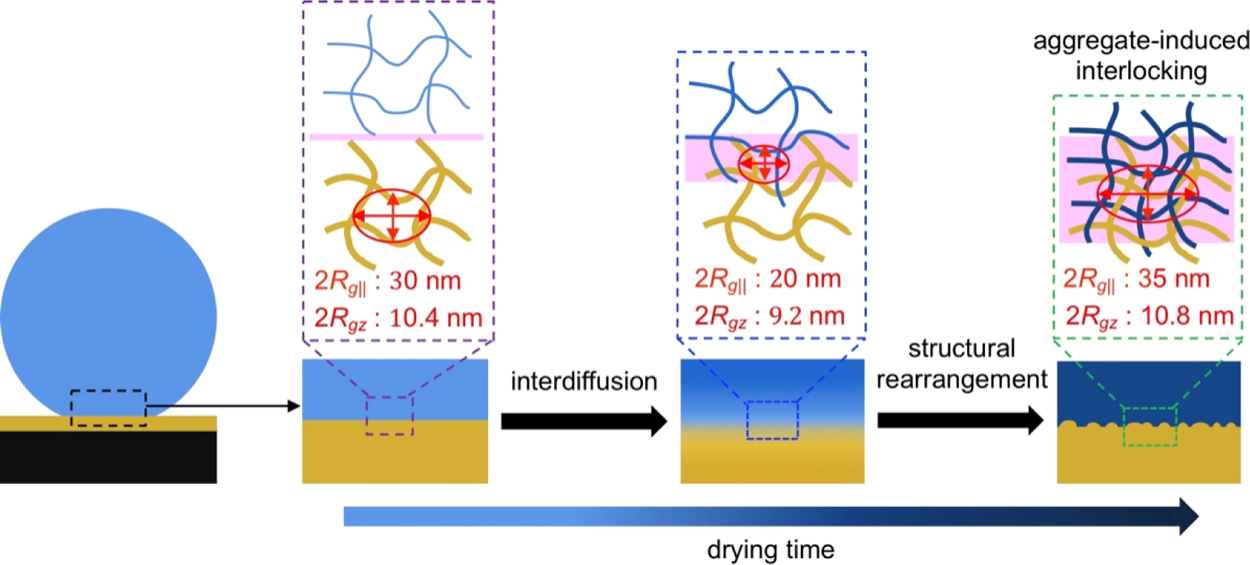
Postulated mechanism behind the development of the adhesive interaction between the cellulose filament and the thin film with the corresponding structural evolution of the interphase during drying.

There are several potential mechanisms that could explain the adhesion of two cellulose surfaces placed in wet contact with each other as they dry. These include, but are not limited to, mechanical interlocking, interdiffusion, hydrogen bonding, induced dipoles, and electrostatic interactions.^19^ It is still not fully clear which mechanism dominates in the interphase between the cellulose beads or filaments and the cellulose thin films. Likewise, it is not known what drives the adhesion between wet, macroscopic, cellulose-rich fibers, and the properties of the dried joints between these fibers.^18,19^ High-resolution techniques such as those developed in this study are invaluable for developing our understanding of these cellulose–cellulose interactions. Because the surface charge of the cellulose used in the present work is very low, the electrostatic interaction can safely be neglected. It is important to mention that hydrogen bonding has basically no influence in the making of the wet contact, where the long-range van der Waals interactions have a much larger influence on bringing the two surfaces together. However, the hydrogen bonding and van der Waals interactions between dried beads and the thin film, where the surfaces are in molecular contact, should be similar for dried water-swollen and ethanol-swollen beads, meaning the joint strength should be the same for dried water-swollen and dried ethanol-swollen beads if they have the same contact area with the thin film after drying, as the joint strength is the area over which the interactions are occurring, times the sum of the different interactions over this area. Because a large difference in pull-off stress σ_s_ was observed for dried water-swollen and ethanol-swollen beads, the molecular contact areas between the cellulose beads and the thin film are much different after drying. Therefore, there must be an additional reason for increasing the molecular contact area, which is the mechanism behind the detected differences in adhesive interactions between the materials. Interestingly, a rougher surface is observed on the dried beads close to, but outside of, the contact area (Figure 4f). This is most probably due to capillary forces, which will deform the surface of the soft swollen bead, pushing it to the thin film surface. However, no cellulose from the thin film was attached to the bead surface (Figure 4f), and no cellulose from the bead was found on the thin film at the position shown in Figure 4j. It can therefore be concluded that (1) a direct mechanical interlocking mechanism between the two surfaces cannot be the main contributing factor to their adhesion and (2) interdiffusion of mobile cellulose molecules in the early drying phase is not sufficient to create a dense interphase layer. Therefore, we propose that, in addition to molecular interdiffusion, there is a structural rearrangement in the later drying phase, which increases the molecular contact area of the two surfaces. This will naturally increase the strength of the joint between the materials. This structural rearrangement is vital for the creation of a larger interphase layer between the two drying surfaces, as depicted in Figure 5. The cellulose molecular chains in the wet filament or bead will slowly diffuse into the cellulose thin film in the early drying phase. This process is then suddenly followed by the creation of aggregated structures after drying for approximately 63 min, according to μGISAXS results (Figure 2). The formation of aggregate structures allows for the creation of more interlocked structures, which, in turn, increases the molecular contact area and leads to the increase of adhesion between the two macroscopic bodies. This model is consistent with the established theory that the fibrils on a macroscopic fiber surface play a decisive role in the strength of the cellulose fiber–fiber joints.^56^

## Conclusions

Although it is not possible to directly verify the molecular interdiffusion with μGISAXS, we have presented a new analytical perspective for tracking the nanoscale structural evolution and interactions at cellulose–cellulose interfaces using μGISAXS and AFM. We have demonstrated that it is possible to correlate the macroscopic mechanical properties with the nanoscale supramolecular structural evolution. We have shown that the strong adhesion between the two noncrystalline cellulose surfaces is mainly due to the molecular interdiffusion and structural rearrangement of cellulose at the interface. To obtain a complete understanding of the complex interactions between cellulose surfaces and their impact on the properties of cellulose-based materials, more work is required. We believe that this study provides a new strategy to prompt further work to enhance our understanding of the solid/solid, solid/liquid, or liquid/liquid interfacial structure between different materials.

## Supporting Information Available

Sample system description of the μGISAXS measurements, μGISAXS data analysis, Guinier-Porod fitting, and SEM images (PDF)

## References

[ref1] Wang S.; Lu A.; Zhang L. Recent Advances in Regenerated Cellulose Materials. Prog. Polym. Sci. 2016, 53, 169–206. 10.1016/j.progpolymsci.2015.07.003.

[ref2] Wang H.; Gurau G.; Rogers R. D. Ionic Liquid Processing of Cellulose. Chem. Soc. Rev. 2012, 41, 1519–1537. 10.1039/c2cs15311d. 22266483

[ref3] Mohanty A. K.; Misra M.; Drzal L. T. Sustainable Bio-Composites from Renewable Resources: Opportunities and Challenges in the Green Materials World. J. Polym. Environ. 2002, 10, 19–26. 10.1023/a:1021013921916.

[ref4] Håkansson K. M. O.; Fall A. B.; Lundell F.; Yu S.; Krywka C.; Roth S. V.; Santoro G.; Kvick M.; Wittberg L. P.; Wågberg L.; Söderberg L. D. Hydrodynamic Alignment and Assembly of Nanofibrils Resulting in Strong Cellulose Filaments. Nat. Commun. 2014, 5, 1–10. 10.1038/ncomms5018. PMC4059937 24887005

[ref5] Mittal N.; Benselfelt T.; Ansari F.; Gordeyeva K.; Roth S. V.; Wågberg L.; Söderberg L. D. Ion-Specific Assembly of Strong, Tough, and Stiff Biofibers. Angew. Chem., Int. Ed. 2019, 58, 18562–18569. 10.1002/anie.201910603. PMC6916401 31600016

[ref6] Moon R. J.; Martini A.; Nairn J.; Simonsen J.; Youngblood J. Cellulose Nanomaterials Review: Structure, Properties and Nanocomposites. Chem. Soc. Rev. 2011, 40, 3941–3994. 10.1039/c0cs00108b. 21566801

[ref7] Postek M. T.; Vladár A.; Dagata J.; Farkas N.; Ming B.; Wagner R.; Raman A.; Moon R. J.; Sabo R.; Wegner T. H.; Beecher J. Development of the Metrology and Imaging of Cellulose Nanocrystals. Meas. Sci. Technol. 2011, 22, 24005 10.1088/0957-0233/22/2/024005.

[ref8] Habibi Y.; Lucia L. A.; Rojas O. J. Cellulose Nanocrystals: Chemistry, Self-Assembly, and Applications. Chem. Rev. 2010, 110, 3479–3500. 10.1021/cr900339w. 20201500

[ref9] Tsioptsias C.; Stefopoulos A.; Kokkinomalis I.; Papadopoulou L.; Panayiotou C. Development of Micro- and Nano-Porous Composite Materials by Processing Cellulose with Ionic Liquids and Supercritical CO2. Green Chem. 2008, 10, 965–997. 10.1039/b803869d.

[ref10] Li T.; Chen C.; Brozena A. H.; Zhu J. Y.; Xu L.; Driemeier C.; Dai J.; Rojas O. J.; Isogai A.; Wågberg L.; Hu L. Developing Fibrillated Cellulose as a Sustainable Technological Material. Nature 2021, 590, 47–56. 10.1038/s41586-020-03167-7. 33536649

[ref11] Klemm D.; Philipp B.; Heinze T.; Heinze U.; Wagenknecht W. Comprehensive Cellulose Chemistry: Volume I: Fundamentals and Analytical Methods; Wiley-VCH Verlag GmbH, 1998; Vol. l.

[ref12] Gericke M.; Trygg J.; Fardim P. Functional Cellulose Beads: Preparation, Characterization, and Applications. Chem. Rev. 2013, 113, 4812–4836. 10.1021/cr300242j. 23540980

[ref13] Yang Q.; Fukuzumi H.; Saito T.; Isogai A.; Zhang L. Transparent Cellulose Films with High Gas Barrier Properties Fabricated from Aqueous Alkali/Urea Solutions. Biomacromolecules 2011, 12, 2766–2771. 10.1021/bm200766v. 21657790

[ref14] Qi H.; Cai J.; Zhang L.; Kuga S. Properties of Films Composed of Cellulose Nanowhiskers and a Cellulose Matrix Regenerated from Alkali/Urea Solution. Biomacromolecules 2009, 10, 1597–1602. 10.1021/bm9001975. 19415903

[ref15] Qi H.; Chang C.; Zhang L. Properties and Applications of Biodegradable Transparent and Photoluminescent Cellulose Films Prepared via a Green Process. Green Chem. 2009, 11, 177–184. 10.1039/b814721c.

[ref16] Müller B.; Gebert-germ M.; Russler A. Viscont HT - the Future of High Performance Viscose Filaments and Their Textile Applications. Lezinger ber. 2012, 90, 64–71.

[ref17] Perepelkin K. E. Lyocell Fibres Based on Direct Dissolution of Cellulose in N-Methylmorpholine N-Oxide: Development and Prospects. Fibre Chem. 2007, 39, 163–172. 10.1007/s10692-007-0032-9.

[ref18] Hirn U.; Schennach R. Fiber-Fiber Bond Formation and Failure: Mechanisms and Analytical Techniques. Proceedings of the 16th fundamental research symposium; Graz University of Technology, 2017; pp 839–863.

[ref19] Lindström T.; Wågberg L.; Larsson T. On the Nature of Joint Strength in Paper—a Review of Dry and Wet Strength Resins Used in Paper Manufacturing. 13th fundamental research symposium; The Pulp and Paper Fundamental Research Society Cambridge, 2005; Vol. 32, pp 457–562.

[ref20] Adamson A. W.; Gast A. P. Physical Chemistry of Surfaces; Interscience publishers: New York, 1967; Vol. 150.

[ref21] Li H.; Kruteva M.; Mystek K.; Dulle M.; Ji W.; Pettersson T.; Wågberg L. Macro- And Microstructural Evolution during Drying of Regenerated Cellulose Beads. ACS Nano 2020, 14, 6774–6784. 10.1021/acsnano.0c00171. 32383585 PMC7315634

[ref22] Li H.; Mystek K.; Wågberg L.; Pettersson T. Development of Mechanical Properties of Regenerated Cellulose Beads during Drying as Investigated by Atomic Force Microscopy. Soft Matter 2020, 16, 6457–6462. 10.1039/d0sm00866d. 32583840

[ref23] Karlsson R.-M. P.; Larsson P. T.; Hansson P.; Wågberg L. Thermodynamics of the Water-Retaining Properties of Cellulose-Based Networks. Biomacromolecules 2019, 20, 1603–1612. 10.1021/acs.biomac.8b01791. 30817883

[ref24] Karlsson R.-M. P.; Larsson P. T.; Yu S.; Pendergraph S. A.; Pettersson T.; Hellwig J.; Wågberg L. Carbohydrate Gel Beads as Model Probes for Quantifying Non-Ionic and Ionic Contributions behind the Swelling of Delignified Plant Fibers. J. Colloid Interface Sci. 2018, 519, 119–129. 10.1016/j.jcis.2018.02.052. 29486431

[ref25] Carrick C.; Pendergraph S. A.; Wågberg L. Nanometer Smooth, Macroscopic Spherical Cellulose Probes for Contact Adhesion Measurements. ACS Appl. Mater. Interfaces 2014, 6, 20928–20935. 10.1021/am505673u. 25382855

[ref26] Levine J. R.; Cohen J. B.; Chung Y. W.; Georgopoulos P. Grazing-Incidence Small-Angle X-Ray Scattering: New Tool for Studying Thin Film Growth. J. Appl. Crystallogr. 1989, 22, 528–532. 10.1107/s002188988900717x.

[ref27] Pietra F.; Rabouw F. T.; Evers W. H.; Byelov D. V.; Petukhov A. V.; de Mello Donegá C.; Vanmaekelbergh D. Semiconductor Nanorod Self-Assembly at the Liquid/Air Interface Studied by in Situ GISAXS and Ex Situ TEM. Nano Lett. 2012, 12, 5515–5523. 10.1021/nl302360u. 23038984

[ref28] Wu L.; Wang X.; Wang G.; Chen G. In Situ X-Ray Scattering Observation of Two-Dimensional Interfacial Colloidal Crystallization. Nat. Commun. 2018, 9, 1–8. 10.1038/s41467-018-03767-y. 29626195 PMC5889402

[ref29] Renaud G.; Lazzari R.; Revenant C.; Barbier A.; Noblet M.; Ulrich O.; Leroy F.; Jupille J.; Borensztein Y.; Henry C. R.; Deville J. P.; Scheurer F.; Mane-Mane J.; Fruchart O. Real-Time Monitoring of Growing Nanoparticles. Science 2003, 300, 1416–1419. 10.1126/science.1082146. 12775836

[ref30] Bommel S.; Kleppmann N.; Weber C.; Spranger H.; Schäfer P.; Novak J.; Roth S. V.; Schreiber F.; Klapp S. H.; Kowarik S. Unravelling the Multilayer Growth of the Fullerene C 60 in Real Time. Nat. Commun. 2014, 5, 5388 10.1038/ncomms6388. 25369851 PMC4272254

[ref31] Hexemer A.; Müller-Buschbaum P. Advanced Grazing-Incidence Techniques for Modern Soft-Matter Materials Analysis. IUCrJ 2015, 2, 106–125. 10.1107/s2052252514024178. PMC4285885 25610632

[ref32] Gu X.; Gunkel I.; Hexemer A.; Gu W.; Russell T. P. An in Situ Grazing Incidence X-Ray Scattering Study of Block Copolymer Thin Films during Solvent Vapor Annealing. Adv. Mater. 2014, 26, 273–281. 10.1002/adma.201302562. 24282077

[ref33] Modestino M. A.; Kusoglu A.; Hexemer A.; Weber A. Z.; Segalman R. A. Controlling Nafion Structure and Properties via Wetting Interactions. Macromolecules 2012, 45, 4681–4688. 10.1021/ma300212f.

[ref34] Brett C. J.; Montani S.; Schwartzkopf M.; van Benthem R. A. T. M.; Jansen J. F. G. A.; Griffini G.; Roth S. V.; Johansson M. K. G. Revealing Structural Evolution Occurring from Photo-Initiated Polymer Network Formation. Chem. Commun. 2020, 3, 1–7. 10.1038/s42004-020-0335-9. PMC9814663 36703468

[ref35] Rossetti F. F.; Panagiotou P.; Rehfeldt F.; Schneck E.; Dommach M.; Funari S. S.; Timmann A.; Müller-Buschbaum P.; Tanaka M. Structures of Regenerated Cellulose Films Revealed by Grazing Incidence Small-Angle x-Ray Scattering. Biointerphases 2008, 3, 117–127. 10.1116/1.3068692. 20408708

[ref36] Ohm W.; Rothkirch A.; Pandit P.; Körstgens V.; Müller-Buschbaum P.; Rojas R.; Yu S.; Brett C. J.; Söderberg D. L.; Roth S. V. Morphological Properties of Airbrush Spray-Deposited Enzymatic Cellulose Thin Films. J. Coating Technol. Res. 2018, 15, 759–769. 10.1007/s11998-018-0089-9.

[ref37] Brett C. J.; Mittal N.; Ohm W.; Gensch M.; Kreuzer L. P.; Körstgens V.; Månsson M.; Frielinghaus H.; Müller-Buschbaum P.; Söderberg L. D.; Roth S. V. Water-Induced Structural Rearrangements on the Nanoscale in Ultrathin Nanocellulose Films. Macromolecules 2019, 52, 4721–4728. 10.1021/acs.macromol.9b00531.

[ref38] Gunnars S.; Wågberg L.; Cohen Stuart M. A. Model Films of Cellulose: I. Method Development and Initial Results. Cellulose 2002, 9, 239–249. 10.1023/a:1021196914398.

[ref39] Eriksson M.; Notley S. M.; Wågberg L. Cellulose Thin Films: Degree of Cellulose Ordering and Its Influence on Adhesion. Biomacromolecules 2007, 8, 912–919. 10.1021/bm061164w. 17319721

[ref40] Benselfelt T.; Pettersson T.; Wågberg L. Influence of Surface Charge Density and Morphology on the Formation of Polyelectrolyte Multilayers on Smooth Charged Cellulose Surfaces. Langmuir 2017, 33, 968–979. 10.1021/acs.langmuir.6b04217. 28045539

[ref41] Larsson P. T.; Svensson A.; Wågberg L. A New, Robust Method for Measuring Average Fibre Wall Pore Sizes in Cellulose I Rich Plant Fibre Walls. Cellulose 2013, 20, 623–631. 10.1007/s10570-012-9850-x.

[ref42] Carrick C.; Ruda M.; Pettersson B.; Larsson P. T.; Wågberg L. Hollow Cellulose Capsules from CO2 Saturated Cellulose Solutions - Their Preparation and Characterization. RSC Adv. 2013, 3, 2462–2469. 10.1039/c2ra22020b.

[ref43] Berthold F.; Gustafsson K.; Berggren R.; Sjöholm E.; Lindström M. Dissolution of Softwood Kraft Pulps by Direct Derivatization in Lithium Chloride/N,N-Dimethylacetamide. J. Appl. Polym. Sci. 2004, 94, 424–431. 10.1002/app.20697.

[ref44] Zhernenkov M.; Canestrari N.; Chubar O.; DiMasi E. Soft Matter Interfaces Beamline at NSLS-II: Geometrical Ray-Tracing vs. Wavefront Propagation Simulations. Advances in Computational Methods for X-Ray Optics III; International Society for Optics and Photonics, 2014; Vol. 9209, p 92090G.

[ref45] Lenz S.; Bonini M.; Nett S. K.; Lechmann M. C.; Emmerling S. G. J.; Kappes R. S.; Memesa M.; Timmann A.; Roth S. V.; Gutmann J. S. Global Scattering Functions: A Tool for Grazing Incidence Small Angle X-Ray Scattering (GISAXS) Data Analysis of Low Correlated Lateral Structures. Eur. Phys. J.: Appl. Phys. 2010, 51, 10601 10.1051/epjap/2010064.

[ref46] Träger A.; Klein G.; Carrick C.; Pettersson T.; Johansson M.; Wågberg L.; Pendergraph S. A.; Carlmark A. Macroscopic Cellulose Probes for the Measurement of Polymer Grafted Surfaces. Cellulose 2019, 26, 1467–1477. 10.1007/s10570-018-2196-2.

[ref47] Gustafsson E.; Johansson E.; Wågberg L.; Pettersson T. Direct Adhesive Measurements between Wood Biopolymer Model Surfaces. Biomacromolecules 2012, 13, 3046–3053. 10.1021/bm300762e. 22924973

[ref48] Roth S. V.; Müller-Buschbaum P.; Timmann A.; Perlich J.; Gehrke R. Structural Changes in Gradient Colloidal Thin Gold Films Deposited from Aqueous Solution. J. Appl. Crystallogr. 2007, 40, s346–s349. 10.1107/s0021889807007716.

[ref49] Yoneda Y. Anomalous Surface Reflection of X Rays. Phys. Rev. 1963, 131, 2010–2013. 10.1103/physrev.131.2010.

[ref50] Schaffer C. J.; Palumbiny C. M.; Niedermeier M. A.; Jendrzejewski C.; Santoro G.; Roth S. V.; Müller-Buschbaum P. A Direct Evidence of Morphological Degradation on a Nanometer Scale in Polymer Solar Cells. Adv. Mater. 2013, 25, 6760–6764. 10.1002/adma.201302854. 24027092

[ref51] Schwartzkopf M.; Santoro G.; Brett C. J.; Rothkirch A.; Polonskyi O.; Hinz A.; Metwalli E.; Yao Y.; Strunskus T.; Faupel F.; Müller-Buschbaum P.; Roth S. V. Real-Time Monitoring of Morphology and Optical Properties during Sputter Deposition for Tailoring Metal-Polymer Interfaces. ACS Appl. Mater. Interfaces 2015, 7, 13547–13556. 10.1021/acsami.5b02901. 26030314

[ref52] Hammouda B. New Guinier-Porod Model. J. Appl. Crystallogr. 2010, 43, 716–719. 10.1107/s0021889810015773.

[ref53] Sartori S.; Knudsen K. D. Small Angle Neutron Scattering. In Neutron Scattering and Other Nuclear Techniques for Hydrogen in Materials; Fritzsche H., Huot J., Fruchart D., Eds.; Springer International Publishing: Cham, 2016; pp 159–191.

[ref54] Israelachvili J. N. Intermolecular and Surface Forces; Academic press, 2015.

[ref55] Luengo G.; Pan J.; Heuberger M.; Israelachvili J. N. Temperature and Time Effects on the “Adhesion Dynamics” of Poly(Butyl Methacrylate) (PBMA) Surfaces. Langmuir 1998, 14, 3873–3881. 10.1021/la971304a.

[ref56] Sugawara E.; Nikaido H. Properties of AdeABC and AdeIJK Efflux Systems of Acinetobacter Baumannii Compared with Those of the AcrAB-TolC System of Escherichia Coli. Antimicrob. Agents Chemother. 2014, 58, 7250–7257. 10.1128/aac.03728-14. 25246403 PMC4249520

