# Supplementary material for: Structure Development of the Interphase between Drying Cellulose Materials Revealed by In Situ Grazing-Incidence Small-Angle X-ray Scattering
Source: Biomacromolecules. 2021 Sep 20;22(10):4274–83. doi: 10.1021/acs.biomac.1c00845 (PMC8512666; doi:10.1021/acs.biomac.1c00845)
Supplement: Supplementary file 1 — bm1c00845_si_001.pdf [file bm1c00845_si_001.pdf]

Supporting Information

Structure Development of the Interphase between Drying Cellulose Materials Revealed by In Situ Grazing-Incidence Small-Angle X-ray Scattering

Hailong Li[1,2], Stephan V. Roth[3], Guillaume Freychet[4], Mikhail Zhernenkov[4], Nadia Asta[1], Lars Wågberg[1,5], and Torbjörn Pettersson[1,5]**

[1]Department of Fibre and Polymer Technology, KTH Royal Institute of Technology, Teknikringen 58, SE-100 44 Stockholm, Sweden

[2]Department of Physics, AlbaNova University Center, Stockholm University, 10691 Stockholm, Sweden

[3]Deutsches Elektronen-Synchrotron (DESY), Notkestr. 85, 22607 Hamburg, Germany

[4]National Synchrotron Light Source II, Brookhaven National Laboratory, Upton, NY 11973, USA

[5]Wallenberg Wood Science Centre, Department of Fibre and Polymer Technology, KTH Royal Institute of Technology, Teknikringen 56, 10044 Stockholm, Sweden

*Correspondence to: haili@kth.se, torbj@kth.se

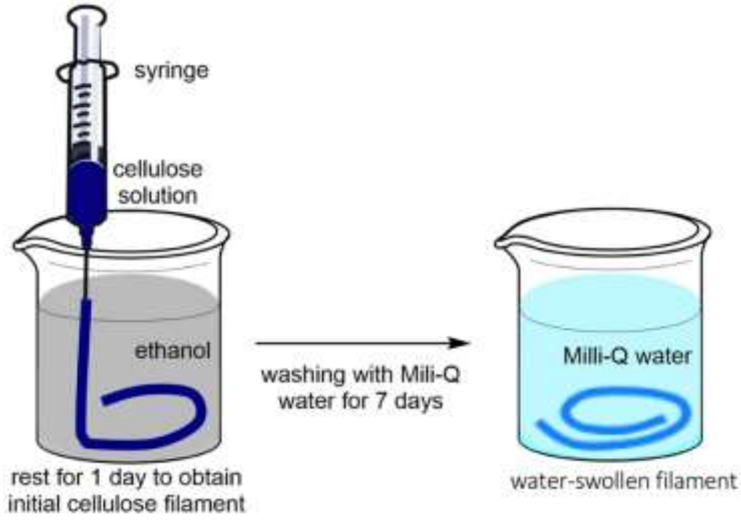

Figure S1. Schematic illustration of the preparation of water-swollen cellulose filament by precipitating a cellulose solution into ethanol, then washing with Milli-Q water for 7 days.

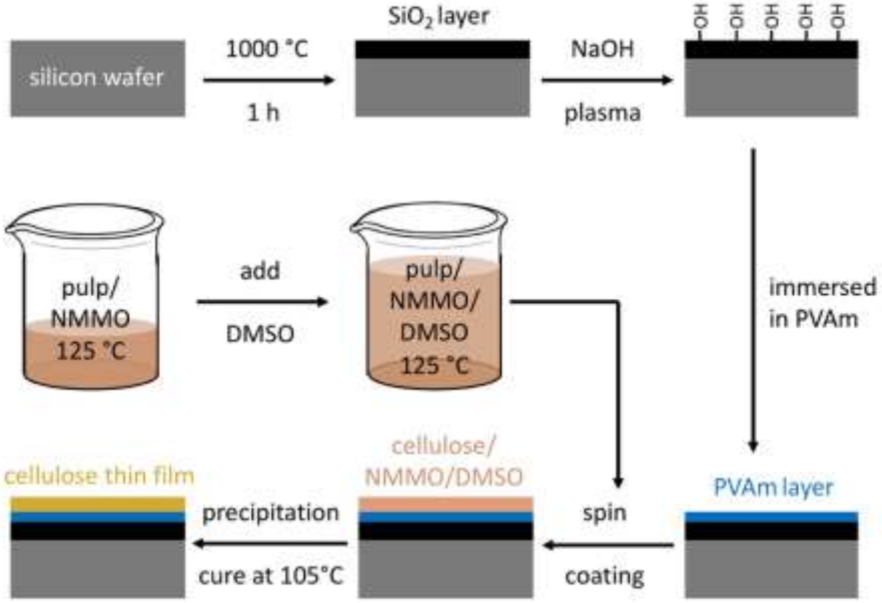

Figure S2. Schematic illustration of the experimental procedure for the preparation of a cellulose thin film.

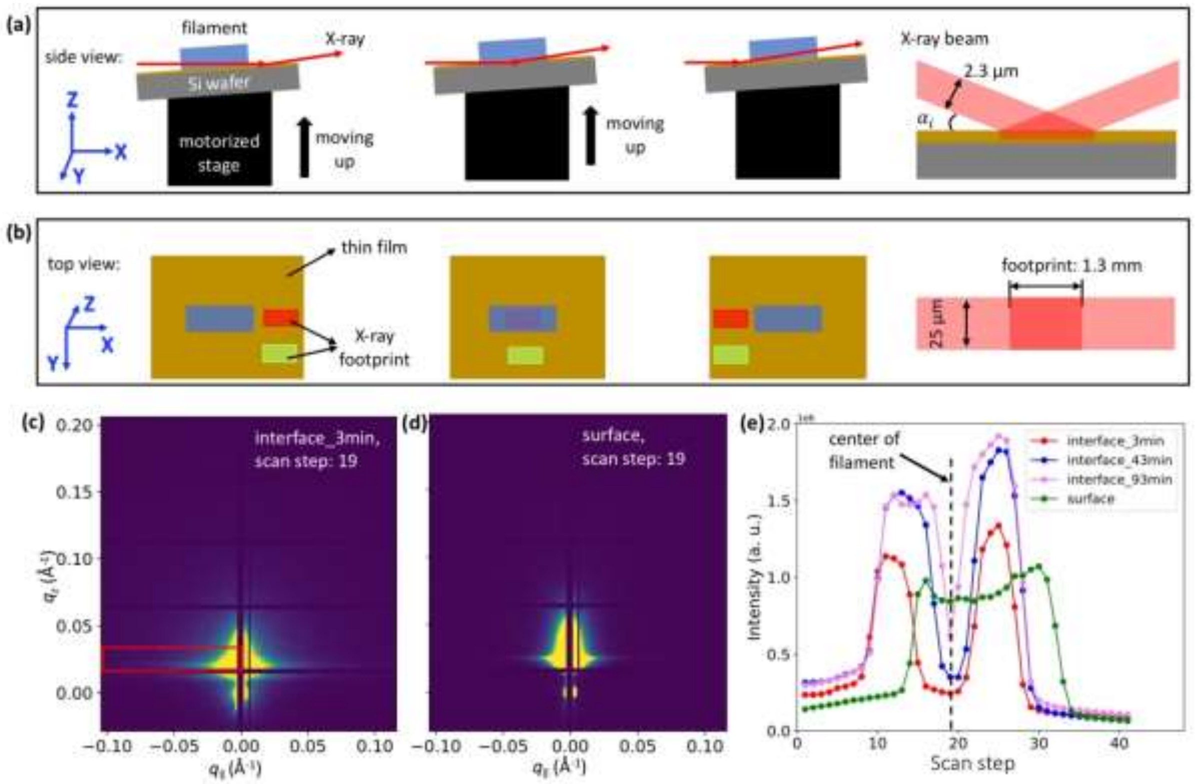

Figure S3. (a) side view and (b) top view of the µGISAXS measurements during Z-scanning. The direction of synchrotron X-ray beam is fixed along the X direction and parallel to the ground surface. The sample was tilted 0.1° to set the X-ray incident angle to be $\alpha_i = 0.1^{o}$. The length of the incident beam footprint was calculated to be: 2.3 µm/*sin*(0.1°)= 1.3 mm. The motorized stage moved relatively from -20 µm to 20 µm with 41 steps in the Z direction, and the X-ray footprint scanned over the whole sample from right to left in the X direction. The red and light green rectangular areas in (b) represent the X-ray footprint position on the interface and surface, respectively. One of the 2D µGISAXS patterns for the (c) interface and (d) surface during Z-scanning. (e) Intensity *vs.* scan step curves for surface and interface at different drying time. The 41 intensity values on each curve were integrated within the red box in C for all 41 2D µGISAXS patterns obtained during the Z-scanning.

Sample system description of the μGISAXS measurements

As illustrated in Figure S4a, before the deposition of the water-swollen gel filament on the dried cellulose thin film, the sample stack can be described as a standard thin film multilayer. The observed peak 2 at q_z = 0.0253 $Å^{-1}$ in Figure 1g (red curve) can be assigned as the Yoneda peak of the dried cellulose thin film. As the corresponding critical angle for peak 2 (α_c= 0.0881°, Figure S5a), calculated from the equation $q = (4\pi \sin\alpha)/\lambda$, is very close to the critical angle of a spray-coated cellulose nanofibril thin film (0.092°) which is converted from the value $\alpha_{c,cellulose}$ = 0.117° with the X-ray energy (E= 13.1 keV).[1,2]

After placing the water-swollen cellulose filament onto the thin film (Figure S4b), the Scattering Length density (SLD) of the gel filament becomes the top subphase, as the x-rays penetrate the near-infinite thickness of the filament from its bottom, which makes it an effective subphase. The consequence is that the q_z profile in Yoneda region changes significantly (Figure 1g), thus, the Yoneda peaks and critical angles need to be calculated relative to this subphase using the equation:

$$\alpha_c = \lambda\sqrt{\frac{SLD - SLD_{subphase}}{\pi}} \quad \text{(s1)}$$

As the cellulose content in the filament increases with drying time, it is anticipated that the SLD of the filament should be in between the SLD of water (9.4×10^{-6} $Å^{-2}$) and the dried thin film (12.5×10^{-6} $Å^{-2}$, calculated from its critical angle 0.0881°). Before drying, the cellulose content in the filament is slightly higher than 1.5 wt%. Thus, the SLD of the gel filament should be larger than $(9.4 \times 0.985 + 12.5 \times 0.015)$ 10^{-6} $Å^{-2}$ = 9.45×10^{-6} $Å^{-2}$. Therefore, the SLD of wet thin film *vs* filament becomes smaller than 3.05×10^{-6} $Å^{-2}$. The critical angle at α_c = 0.04° observed on Figure S5b corresponds to SLD of 2.6×10^{-6} $Å^{-2}$. Indicating that this small peak is from the wet cellulose thin film. However, it is too weak and disappears during drying, thus, we do not track its change

with drying time. Moreover, the SLD of SiO_2 layer *vs* filament coincides with the SLD of the dried thin film, that's the reason why the Yoneda peak 2 is still observed but shifts to lower q. The newly appeared peak at q_z = 0.0225 $Å^{-1}$ (peak 1 in Figure 1g) is most probably due to the diffusion of the water molecules and cellulose molecular chains from the outermost layer of gel filament into the thin film (Figure S4b), which will cause the dried thin film to swell. This is consistent with the sharp decrease of $d_{\|}$ at the same drying time in Figure 1f.

After filament drying for 2 hours, a denser and rougher layer is formed at the interface, named the "interphase layer" (Figure S4c). This is based on the observation that peak 2 (pink and purple curves in Figure 1g) become broader and the intensity-drop shifts to higher q_z comparing to the dry thin film surface. This might be caused by the structural rearrangement of cellulose chains in the interphase layer in the later drying phase.

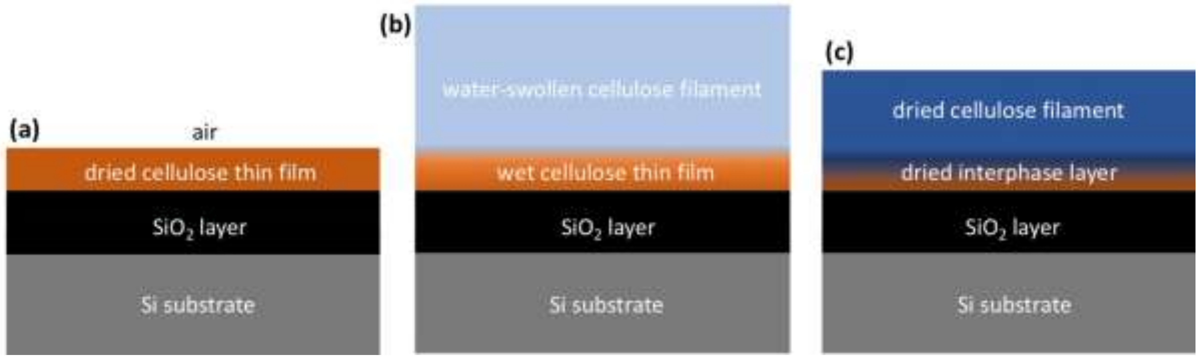

Figure S4. Sample system for the μGISAXS. (a) before placing the gel filament on the cellulose thin film. (b) after placing the gel filament on the cellulose thin film. (c) after drying the filament.

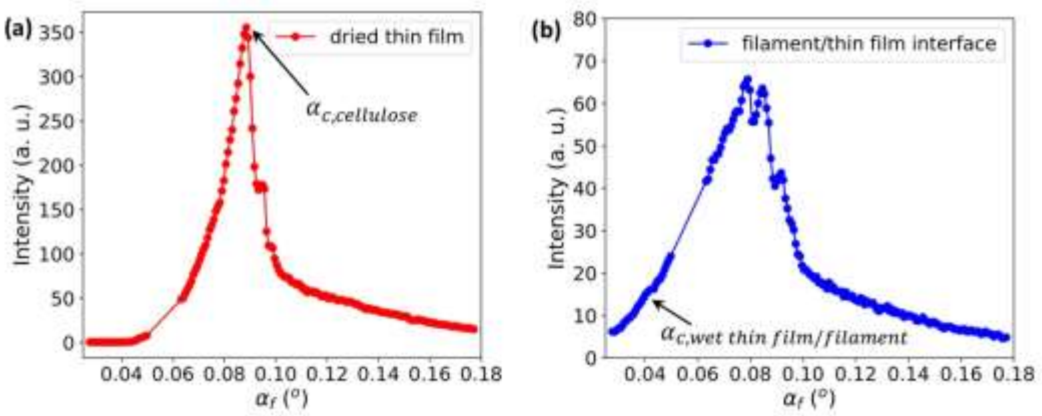

Figure S5. The Yoneda peaks in the vertical cuts of μGISAXS data for (a) the dried thin film surface and (b) the filament/thin film interface after drying for 3 min.

Table S1. Scattering Length densities (SLD) and the corresponding critical angle (α_c) for different materials. SLD of the filament is calculated for the materials before drying starts.

Material	subphase	SLD (10^{-6} A^{-2})	α_c (°)
Si	air	19.84	1.12E-01
SiO_2		22.4	1.19E-01
Dried thin film		12.5	8.81E-02[a]
water		9.4	7.63E-02
filament		9.45	7.66E-02
SiO_2	filament	12.95	8.96E-02
Wet thin film		2.6	4.01E-02[b]

[a, b] values are obtained from Figure S5.

μGISAXS data analysis

A Guinier-Porod empirical model[3] was used to fit both vertical and horizontal cuts. This model assumes a characteristic length scale in the system defined as R_g, and a 'dimensionality' parameter (n) to characterize the shape of the corresponding scattering object. It models the form factor for nonspherical objects with the following functional forms:

$$I(q) = \mathrm{G}\, exp\left(\frac{-q^2R_g^2}{3}\right) \text{ for } q \leq q_1, \tag{s2}$$

$$I(q) = \frac{D}{q^n} \text{ for } q \geq q_1. \tag{s3}$$

where q is the scattering vector, $I(q)$ is the scattering intensity, R_g is the radius of gyration, n is the Porod exponent, and G and D are the Guinier and Porod scale factors, respectively. With the requirement that the values of the Guinier and Porod terms and their derivatives be continuous at q_1, the following relationships are obtained:

$$q_1 = \frac{1}{R_g}\left[\frac{3n}{2}\right]^{\frac{1}{2}}, \tag{s4}$$

$$D = G\, exp\left(\frac{-q_1^2R_g^2}{3}\right) q_1^n = G\, exp\left(-\frac{n}{2}\right)\left(\frac{3n}{2}\right)^{n/2}\frac{1}{R_g^n}. \tag{s5}$$

During fitting, the Guinier form is used for $q \leq q_1$ and the Porod form is used for $q \geq q_1$. The two forms are never used concurrently.

Representative vertical and horizontal cuts of μGISAXS data and the corresponding fitting curves were plotted in Figure S6. It is observed that this model is sufficient to fit both vertical and horizontal cuts of the μGISAXS data. Moreover, as discussed by Lenz *et al.*,[4] fitting R_{gz} while neglecting the Distorted-Wave Born Approximation (DWBA) reflection terms only enables an

extraction of an approximate value of R_{gz}. However, it is reliable enough to indicate how the structure changes normal to the surface.

In this Guinier-Porod model,[3,5] Porod exponents less than 3 are for 'mass fractals'. A Porod exponent $n = 5/3$ points to scattering from 'fully swollen' polymer chains (in a good solvent) and $n = 3$ points to 'collapsed' chains (in a bad solvent). While Porod exponents between 3 and 4 are for 'surface fractals'. An exponent $n = 3$ can also point to particles with rough surfaces and $n = 4$ points to very smooth surfaces.

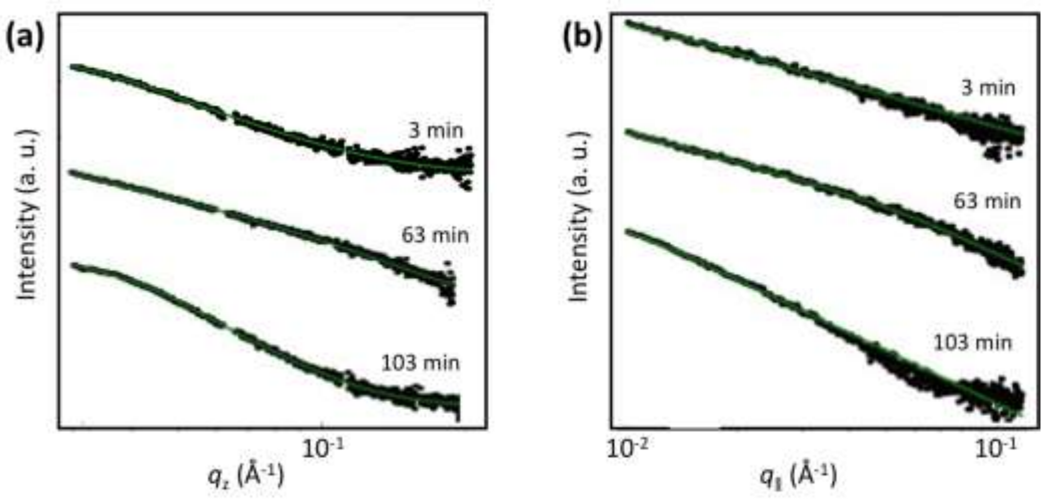

Figure S6. Guinier-Porod fitting. (a) Vertical cuts of µGISAXS data for the filament/thin film interface after drying for 3, 63, and 103 min (black points) and the corresponding fitting curves in green. (b) Horizontal cuts of µGISAXS data for the filament/thin film interface after drying for 3, 63, and 103 min (black points) and the corresponding fitting curves in green.

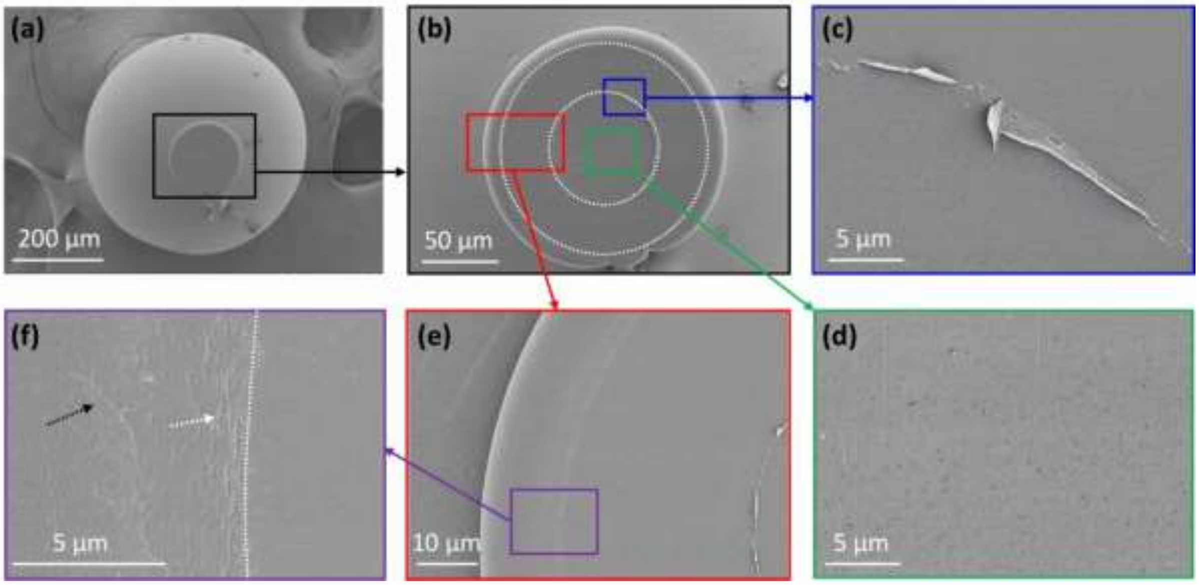

Figure S7. (a) SEM image of the bottom of the dried ethanol-swollen bead. (b-f) The corresponding high magnification SEM images in the boxes.

References

(1) Brett, C. J.; Mittal, N.; Ohm, W.; Gensch, M.; Kreuzer, L. P.; Körstgens, V.; Månsson, M.; Frielinghaus, H.; Müller-Buschbaum, P.; Söderberg, L. D.; Roth, S. V. Water-Induced Structural Rearrangements on the Nanoscale in Ultrathin Nanocellulose Films. *Macromolecules* **2019**, *52*, 4721–4728.

(2) Ohm, W.; Rothkirch, A.; Pandit, P.; Körstgens, V.; Müller-Buschbaum, P.; Rojas, R.; Yu, S.; Brett, C. J.; Söderberg, D. L.; Roth, S. V. Morphological Properties of Airbrush Spray-Deposited Enzymatic Cellulose Thin Films. *J. Coatings Technol. Res.* **2018**, *15*, 759–769.

(3) Hammouda, B. A New Guinier-Porod Model. *J. Appl. Crystallogr.* **2010**, *43*, 716–719.

(4) Lenz, S.; Bonini, M.; Nett, S. K.; Lechmann, M. C.; Emmerling, S. G. J.; Kappes, R. S.; Memesa, M.; Timmann, A.; Roth, S. V.; Gutmann, J. S. Global Scattering Functions: A Tool for Grazing Incidence Small Angle X-Ray Scattering (GISAXS) Data Analysis of Low Correlated Lateral Structures. *EPJ Appl. Phys.* **2010**, *51*, 10601.

(5) Sartori, S.; Knudsen, K. D. Small Angle Neutron Scattering. In *Neutron Scattering and Other Nuclear Techniques for Hydrogen in Materials*; Fritzsche, H., Huot, J., Fruchart, D., Eds.; Springer International Publishing: Cham, 2016; pp 159–191.
